# Antidepressants in the treatment of bipolar depression: commentary

**DOI:** 10.1093/ijnp/pyaf013

**Published:** 2025-02-18

**Authors:** Gustavo H Vázquez, Ross J Baldessarini

**Affiliations:** International Consortium for Mood & Psychotic Disorders Research, Mailman Research Center, McLean Hospital, Belmont, MA, United States; Department of Psychiatry, School of Medicine, Queen’s University, Kingston, Ontario, Canada; International Consortium for Mood & Psychotic Disorders Research, Mailman Research Center, McLean Hospital, Belmont, MA, United States; Department of Psychiatry, Harvard Medical School, Boston, MA, United States

**Keywords:** antidepressants, antipsychotics, bipolar disorder, depression, major depressive disorder, mood stabilizers

## Abstract

**Background:**

Depression is a therapeutic challenge with bipolar disorder (BD) patients and remains a major contributor to disability, comorbidity, and premature mortality. The efficacy and safety of antidepressants (ADs) for this indication remain particularly controversial, and optimally safe and effective treatment of bipolar (BP) depression remains uncertain.

**Method:**

We summarized selected research findings on the treatment of depression in BD aimed at supporting practical guidelines for clinical treatment involving ADs.

**Results:**

Growing research evidence indicates that ADs are probably effective in BP depression and possibly not less than in major depressive disorder. Tolerability of antidepressant (AD) treatment is greater with type II BD (BD-2) than with type I (BD-1), particularly when ADs are combined with a mood stabilizer or antipsychotic. For BP depression, preferred ADs are serotonin-reuptake inhibitors and bupropion given in moderate doses for limited times.

**Conclusions:**

Optimal treatment of depression requires further investigation, particularly for long-term maintenance. Nevertheless, treatment for acute depressive episodes can usefully and safely include some ADs in moderate doses for limited duration, best combined with lithium, some anticonvulsants, or certain atypical antipsychotics, and more safely with BD-2 than BD-1 with close clinical supervision.

## INTRODUCTION

Bipolar disorder (BD) is a complex and heterogeneous mood disorder characterized by recurrent episodes of mania, hypomania, mixed states, and depression, often with sustained dysthymia. Psychiatric nosography currently classifies BD into 2 main subtypes: bipolar I disorder (BD-1), marked by the presence of at least one manic and one major depressive episode, and bipolar II disorder (BD-2) with recurrent depressive episodes and at least one nonpsychotic hypomanic episode.^1,2^ The natural course of BD involves a marked predominance of depressive and dysthymic components. Even with clinically appropriate treatment, total morbidity in BD has averaged 44.6% [39.6–49.6] of time at risk, with depressive states accounting for 76% of this unresolved morbidity.^3^ Treatment of bipolar (BP) depression, even with modern therapies, is more difficult than for [hypo]manic phases and remains challenging.^4,5^ Antidepressants (ADs), though commonly prescribed, carry risks, which vary with the type of drug, dose, and subtype of BD.^5,6^

The present commentary aims to summarize the current understanding of antidepressant (AD) use in the treatment of BD patients, considering pros and cons as well as differences in treatment approaches between the 2 major subtypes of BD. It is not a comprehensive review of evidence concerning treatment options for BP depression but instead focuses on specific aspects of AD use for that indication. In addition to ADs, other important treatments for BP depression include mood stabilizers, atypical antipsychotics, electroconvulsive therapy (ECT), and transcranial magnetic stimulation (TMS).^7-9^

## RATIONALE FOR USE OF ADs IN BD

Antidepressants are widely used empirically in managing acute depressive episodes of BD, following the long history of research and practice that established such medicines as the cornerstone of the treatment of nonbipolar forms of major depression.^4,5,10^ The rationale for the use of ADs to treat BP depression arises from the need to alleviate depressive symptoms which significantly impair quality of life and functional capacity and elevate risks of disability as well as of mortality associated with both suicide and co-occurring general medical disorders^5,11,12^ It has been recommended that AD doses for treating patients with BD be lower than for major depressive disorder (MDD), and that duration of treatment should last only to the end of the index depressive episode rather than as a prolonged continuation treatment.^13^ However, both the efficacy and safety of ADs in BD have been subjects of considerable debate, with particular concern about the risk of triggering manic or psychotic episodes and sustained mood destabilization.^6,14^

## EFFICACY OF ADs IN BIPOLAR DEPRESSION

A growing body of research evidence indicates that ADs can be effective in treating depressive episodes in BD. Systematic reviews and meta-analyses indicate that ADs, particularly when used in conjunction with mood stabilizers or second-generation (atypical) antipsychotics, can provide significant benefits in reducing depressive symptoms with low risk of inducing mania^9,13,15-18^ Some clinical trials also have found the efficacy of ADs in BP depression not to be inferior to that observed in nonbipolar MDD^6,14,15,19^ Nevertheless, controversy about the efficacy and safety of AD treatment for BP depression has arisen from some controlled trials that failed to differentiate ADs from placebo or other control conditions as well as concern about risks of adverse outcomes.^14^

A sampling of evidence about efficacy of AD treatment for acute BP depressive episodes includes findings in 10 trials (4/10 with added mood stabilizer or antipsychotic) with response rates of 256/571 (44.8% [95% CI, 40.7–49.0]) with ADs vs 288/861 with placebo (33.4% [30.3–36.7], a 1.34-fold difference; χ^2^ = 18.9, *P* < .0001).^16^ A later review by Baldessarini et al.^6^ of 12 monotherapy trials found response rates of 393/803 (48.9% [45.4–52.5] with ADs vs 419/1092 (38.4% [35.5–41.3]) with placebo, a moderate (1.27-fold) but statistically highly significant difference of 1.27-fold (χ^2^ = 21.1; meta-analytic pooled risk ratio = 1.32 [1.07–1.62]; both *P* < .0001). However, only 3/12 of these trials individually yielded significant superiority of AD over placebo.

A recent comprehensive review^17^ found the following ranking of responses to treatments in acute BP depression: [1] antidepressants + antipsychotics (5 trials, 314 subjects; standardised mean difference [SMD] vs placebo = 0.40 [0.17–0.63]); [2–3] ADs (13 trials, 698 subjects; SMD = 0.28 [0.12–0.43]); [2–3]; atypical antipsychotics (29 trials, 6526 subjects; SMD = 0.28 [0.21–0.35]; [4] anticonvulsants (19 trials, 1149 subjects; SMD = 0.16 [0.04–0.28]). These several findings suggest that ADs alone probably are more effective than placebo for acute BP depression, that their combination with certain antipsychotics (also effective alone) may be even more effective, and that anticonvulsants were less effective. Lithium continues to be remarkably understudied in acute BP depression but has shown little superiority over placebo (response rate with lithium = 85/136 [62.5% (53.8–70.0)] vs an unusually high rate of response with placebo at 72/129 [55.8% (46.8–64.5)]) for a 1.12-fold difference.^6^

## RISKS ASSOCIATED WITH ADs IN BD

Despite its potential benefits, AD treatment in BD is fraught with risks. There is notable concern that ADs may precipitate manic or hypomanic episodes (“switching”), psychosis, or otherwise induce emotionally and behaviorally destabilizing effects. There is particular concern for BD-1 patients with their unique potential for manic and psychotic phases.^1,2,6,14,19-21^ This risk appears to be especially pronounced among patients with a history of mood-switching during previous AD treatment or of a rapid cycling course.^20-22^ Moreover, long-term use of ADs with prophylactic intent raises questions about the potential of such treatment for sustained destabilization and increased cycling between mood states.^22,23^ This practice is not well balanced by research to support long-term depression-preventing effects of ADs even when combined with mood-stabilizing treatments.^20-24^

Another topic of important clinical concern is the effect of including ADs in the treatment of depressive phases of BD on suicidal risk. The claim has been made, based mainly on higher rates of suicidal ideation in individuals suffering from MDD reported among patients under age 25 years, that AD treatment may increase suicidal risk in mood disorder patients.^25,26^

However, the risk of suicidal ideation may be *lower* with AD treatment of MDD patients older than 25 years.^25,26^ Moreover, the relevance of such findings to the safety of AD treatment in BD is uncertain. These uncertainties coupled with the unusually high risks of suicidal behavior associated with BDs call for extra vigilance for emerging circumstances that may be associated with suicides and attempts, including the development of mixed manic-depressive features^6^ and during periods of very high risk soon after discharge from psychiatric hospitalization.^27^ Since lithium is often combined with AD treatment of BD patients, its evidence of reducing risks of suicidal behavior is of particular clinical interest for the overall management of BP depression.^28^

## AD TREATMENT WITH TYPES I VS II BD

### Differences in Clinical Presentation

BD-1 and BD-2 exhibit distinct clinical features that influence treatment approaches. BD-1 is characterized by more severe episodes of mood elevation, sometimes with psychotic features and severe behavioral dyscontrol, which can lead to major functional impairment, dangerous, impulsive behaviors, and require intensive management strategies. In contrast, BD-2 is defined as involving milder (hypomanic) mood elevations without psychosis, though with more frequent and prolonged depressive states, making management of depression the primary focus for the treatment of BD-2.^1,2,29^

### Implications for AD Use

Differences in clinical manifestations of BD-1 and BD-2 have important implications for the use of ADs. In BD-1, the risk of inducing mania is a critical consideration, leading many clinicians to adopt a cautious approach and avoid prescribing ADs, particularly as a monotherapy. It is common for clinicians to prioritize mood stabilizers or second-generation antipsychotics over ADs for this population, particularly with a history of manic episodes, psychotic features, and rapid cycling.^14^ Often such medicines are combined with ADs in the hope of avoiding excessive mood elevation. While the efficacy of this widely accepted practice is highly plausible, evidence from randomized trials that mood-stabilizing agents (such as lithium and certain anticonvulsants) or antipsychotic drugs can prevent mood-switching in AD-treated BD patients is lacking.^6,21^ Moreover, nonrandomized clinical comparisons risk confounding by indication in that patients most in need of mood stabilizers by history are most likely to receive them.^21^ Also, mood stabilizers alone may be less effective than ADs in acute BP depression^17^ and they and antipsychotic drugs carry risks of clinically important adverse neurological and metabolic effects.

BD-2 patients typically experience prolonged depressive episodes and dysthymia.^3,5^ This natural history and lack of risk of switching into mania or psychosis may justify a more aggressive approach to treating depressive symptoms, including relatively liberal use of ADs. Moreover, emerging evidence increasingly supports both the efficacy and safety of AD treatment in BD-2 depression, either alone or with mood stabilizing co-treatment^16,29,30^ It follows that ADs can be employed more liberally for BD-2 depression, especially when used in conjunction with mood stabilizers or certain atypical antipsychotics with the aim of reducing the risk of mood destabilization.^13^ Nevertheless, the body of research support for the safety and efficacy of such practices specifically for BD-2 depression is quite limited^30^, and clinical vigilance with close supervision is appropriate when treating such patients.^31^ Moreover, the efficacy and safety of ADs given with mood stabilizers for long-term maintenance or prophylactic treatment in BD aimed at preventing recurrences of depression remains very little studied.^20,24^

## CLINICAL CONSIDERATIONS AND TREATMENT STRATEGIES

### Monitoring and Patient Selection

When deciding on the use of ADs for BD patients, careful patient selection and adequate clinical monitoring are paramount. Clinicians should assess the patient’s history of mood episodes (considering their predominant polarity, frequency, severity, and complications), previous treatment responses, and the presence of co-occurring psychiatric or general medical conditions that may complicate treatment. Patients with significant mood instability or rapid cycling may be less suitable candidates for AD therapy.^6^

### Combining ADs With Mood stabilizers or Antipsychotics

To enhance the safety and efficacy of AD treatment, clinicians should consider the concurrent use of mood stabilizers (particularly lithium or valproate) or some second-generation antipsychotics preferably those with evidence of beneficial effects in acute BP depression (including cariprazine, lurasidone, lumateperone, olanzapine [best with fluoxetine], and quetiapine) as well as demonstrated or expected benefits in mania, while avoiding first-generation antipsychotics for lack of evidence of efficacy in BP depression and high risks of adverse neurological effects.^6,18,32^ There is also emerging evidence that treatment with long-acting injected preparations of atypical antipsychotic drugs can provide protective effects against recurrences in BD patients,^33^ although their effects in combination with ADs require investigation. The strategy of including a mood-stabilizing treatment with ADs very plausibly aims at providing a buffer against potential mania-inducing risks associated with AD monotherapy, particularly in BD-1. Nevertheless, again, research evidence that such treatments can avoid excessive mood-elevating or destabilizing effects of AD treatment is surprisingly limited.^14,21,34^ Indeed the added risk of mood-switching with an AD added is much smaller than seems to be widely appreciated. Notably, the overall [hypo]manic switch risk with vs without ADs given to BD patients has averaged 15.4% vs 13.7%, a minor 1.12-fold difference.^21^

### Individualized Treatment Approaches

Treatment of BP depression with ADs requires an individualized approach and consideration of the unique characteristics of each patient’s illness, vulnerabilities, and supports. For BD-2 patients, whose depressive episodes are especially challenging, a trial of ADs may be warranted, perhaps particularly coupled with a mood stabilizer. In contrast, for BD-1 patients, a more conservative approach usually is appropriate, emphasizing mood stabilization as the primary goal before considering addition of ADs.^13^

Also important is the choice of AD. The risk of inducing manic switching is much greater with tricyclic ADs as well as venlafaxine and possibly desvenlafaxine than with moderate doses of serotonin-reuptake inhibitors or bupropion.^21^

Moreover, such ADs should be started cautiously at low, slowly increased doses with close clinical monitoring and continued only if and until helpful mood elevation emerges, and then reduced in dose and gradually discontinued in favor of relying on a mood-stabilizing regimen.

## GUIDELINES AND EXPERT CONSENSUS RECOMMENDATIONS

Guidelines from the International Society for Bipolar Disorders^14^ and the Canadian Network for Mood and Anxiety Treatments^13^ recommend using ADs in BD but emphasize caution. Specifically, they suggest that ADs should be considered for BD-2 patients but approached more cautiously with BD-1 depression to avoid the risk of manic or psychotic episodes and sustained destabilization.^13,14^ The American Psychiatric Association^35^ has also emphasized the importance of mood stabilization before initiating AD therapy for BD patients. They recommend that ADs generally should not be used as monotherapy in BP depression, particularly for BD-1 patients with a history of manic or psychotic episodes.^35^

## CURRENT UNKNOWNS AND FUTURE DIRECTIONS

Despite existing guidelines and substantial research efforts, several unknowns remain regarding the use of ADs in BD. The optimal duration of treatment, the precise comparative efficacy and tolerability of different AD types, the relative value of emerging treatments with AD effects such as ketamine and psychedelics, optimal combinations and doses of particular ADs with specific mood stabilizers and antipsychotics, relative benefits and safety of nonpharmacological treatments (such as ECT and repetitive transcranial magnetic stimulation), and long-term safety profiles of all such treatments require investigation. Additionally, the role of psychological and social interventions, including psychotherapy and lifestyle modifications, with pharmacotherapies warrants further exploration^36,37^ and consideration in tailoring treatment plans for individual BD patients.

Future research should focus on large-scale, well-controlled trials to better understand the complex effects of ADs, mood stabilizers, and antipsychotics in both BD-1 and BD-2. These studies also need to investigate the potential benefits of personalized approaches that consider individual patient profiles, including the nature, frequency, and severity of mood episodes and co-occurring psychiatric and general medical disorders.

## CONCLUSIONS

The use of ADs in the management of BDs is a complex and still controversial topic that requires thoughtful planning by clinicians based on available research evidence. While AD medicines can effectively mitigate depressive symptoms in BD patients, their potential risks—particularly induction of mania, psychosis, or destabilization—call for a cautious approach, especially with BD-1 patients. For BD-2 patients, their great excess of potentially disabling and life-threatening depressive morbidity encourages the use of ADs, provided that appropriate safeguards and close clinical supervision are in place and possibly as a supplement.^6^

As understanding of mood disorders continues to evolve, ongoing research is essential to refine treatment protocols and enhance patient outcomes. Clinicians must remain informed about the latest evidence and guidelines to navigate the challenges associated with AD use in BD effectively. By adopting a thoughtful and individualized approach, mental health professionals can optimize treatment strategies and improve the quality of life for individuals living with BD.

## References

[1] Tondo L , Miola A, Pinna M, Contu M, Baldessarini RJ. Differences between bipolar disorder types 1 and 2 support the DSM two-syndrome concept. Int J Bipolar Disord. 2022; 10:21–31. https://doi.org/10.1186/s40345-022-00268-2 PMC9346033 35918560

[2] Hernandorena CV , Baldessarini RJ, Tondo L, Vázquez GH. Status of type II vs. type I bipolar disorder: systematic review with meta-analyses. Harv Rev Psychiatry. 2023; 31:173–182. https://doi.org/10.1097/HRP.0000000000000371 37437249

[3] Forte A , Baldessarini RJ, Tondo L, et al Long-term morbidity in bipolar-I, bipolar-II, and unipolar major depressive disorders. J Affect Disord. 2015; 178:71–78. https://doi.org/10.1016/j.jad.2015.02.011 25797049

[4] Baldessarini RJ. Chemotherapy in Psychiatry. 3rd ed. Springer Press; 2013.

[5] Baldessarini RJ , Vázquez GH, Tondo L. Bipolar depression: a major unsolved challenge. Int J Bipolar Disord. 2020; 8:1–13. https://doi.org/10.1186/s40345-019-0160-1 31903509 PMC6943098

[6] Baldessarini RJ , Tondo L, Vázquez GH. Pharmacological treatment of adult bipolar disorder. Mol Psychiatry. 2019; 24:198–217. https://doi.org/10.1038/s41380-018-0044-2 29679069

[7] Taylor DM , Cornelius V, Smith L, Young AH. Comparative efficacy and acceptability of drug treatments for bipolar depression: a multiple-treatments meta-analysis. Acta Psychiatr Scand. 2014; 130:452–469. https://doi.org/10.1111/acps.12343 25283309

[8] Vázquez GH , Camino S, Tondo L, Baldessarini RJ. Potential novel treatments for bipolar depression: ketamine, fatty acids, anti-inflammatory agents, and probiotics. CNS Neurol Disord Drug Targets. 2017; 16:858–869. https://doi.org/10.2174/1871527316666170728165648 28758582

[9] Bahji A , Ermacora D, Stephenson C, Hawken ER, Vázquez GH. Comparative efficacy and tolerability of pharmacological treatments for the treatment of acute bipolar depression: a systematic review and network meta-analysis. J Affect Disord. 2020; 269:154–184. https://doi.org/10.1016/j.jad.2020.03.030 32339131

[10] Baldessarini RJ , Henk H, Sklar A, Chang J, Leahy L. Psychotropic medications for patients with bipolar disorder in the United States: polytherapy and adherence. Psychiatr Serv. 2008; 59:1175–1183. https://doi.org/10.1176/appi.ps.59.10.1175 18832504

[11] Chen PH , Tsai SY, Chen PY, et al Mood stabilizers and risk of all-cause, natural, and suicide mortality in bipolar disorder: a nationwide cohort study. Acta Psychiatr Scand. 2023; 147:234–247. https://doi.org/10.1111/acps.13519 36367926

[12] Nierenberg AA , Agustini B, Köhler-Forsberg O, et al Diagnosis and treatment of bipolar disorder: a review. JAMA. 2023; 330:1370–1380. https://doi.org/10.1001/jama.2023.18588 37815563

[13] Yatham LN , Kennedy SH, Parikh S, et al Canadian Network for Mood and Anxiety Treatments (CANMAT) and International Society for Bipolar Disorders (ISBD) for the management of patients with bipolar disorder. Bipolar Disord. 2018; 11:229–255. 10.1111/bdi.12609 PMC5947163 29536616

[14] Pacchiarotti I , Bond DJ, Baldessarini RJ, et al International Society for Bipolar Disorders (ISBD) task-force report on antidepressant use in bipolar disorders. Am J Psychiatry. 2013; 170:1249–1262. https://doi.org/10.1176/appi.ajp.2013.13020185 24030475 PMC4091043

[15] Vázquez G , Tondo L, Baldessarini RJ. Comparison of antidepressant responses in patients with bipolar vs. unipolar depression: a meta-analytic review. Pharmacopsychiatry. 2011; 44:21–26. https://doi.org/10.1055/s-0030-1265198 21031345

[16] Vázquez GH , Tondo L, Undurraga J, Baldessarini RJ. Overview of antidepressant treatment of bipolar depression. Int J Neuropsychopharmacol. 2014; 16:1673–1685. https://doi.org/10.1017/s1461145713000023 23428003

[17] Yildiz A , Siafis S, Mavridis D, Vieta E, Leucht S. Comparative efficacy and tolerability of pharmacological interventions for acute bipolar depression in adults: a systematic review and network meta-analysis. Lancet Psychiatry. 2023; 10:693–705. https://doi.org/10.1016/s2215-0366(23)00199-2 37595997

[18] Li S , Xu C, Hu S, Lai J. Efficacy and tolerability of FDA-approved atypical antipsychotics for the treatment of bipolar depression: a systematic review and network meta-analysis. Eur Psychiatry. 2024; 67:e29–e37. https://doi.org/10.1192/j.eurpsy.2024.25 38487836 PMC10988162

[19] Baldessarini RJ , Miola A, Vázquez GH, Tondo L. Responses to clinical treatment of bipolar vs. unipolar depressive episodes in women vs. men. J Psychopharmacol. 2025; 39:171–175. https://doi.org/10.1177/02698811241292946 39511786

[20] Ghaemi SN , Wingo AF, Filkowski MA, Baldessarini RJ. Long-term antidepressant treatment in bipolar disorder: meta-analyses of benefits and risks. Acta Psychiatr Scand. 2010; 118:347–356. 10.1111/j.1600-0447.2008.01257.x PMC2718794 18727689

[21] Tondo L , Vázquez GH, Baldessarini RJ. Mania associated with antidepressant treatment: comprehensive meta-analytic review. Acta Psychiatr Scand. 2010; 121:404–414. https://doi.org/10.1111/j.1600-0447.2009.01514.x 19958306

[22] El-Mallakh RS , Vöhringer PA, Ostacher MM, et al Antidepressants worsen rapid-cycling course in bipolar depression–a STEP-BD randomized clinical trial. J Affect Disord. 2015; 184:318–321. https://doi.org/10.1016/j.jad.2015.04.054 26142612 PMC4519402

[23] Leverich GS , Altshuler LL, Frye MA, et al Risk of switch in mood polarity to hypomania or mania in patients with bipolar depression during acute and continuation trials of venlafaxine, sertraline, and bupropion as adjuncts to mood stabilizers. Am J Psychiatry. 2006; 163:232–239. https://doi.org/10.1176/appi.ajp.163.2.232 16449476

[24] Nestsiarovich A , Gaudio CES, Baldessarini RJ, et al Preventing new episodes of bipolar disorder in adults: systematic review and meta-analysis of randomized controlled trials. Eur Neuropsychopharmacol. 2022; 54:75–89. 34489127 10.1016/j.euroneuro.2021.08.264

[25] Baldessarini RJ , Tondo L, Strombom IM, et al Ecological studies of antidepressant treatment and suicidal risks. Harv Rev Psychiatry. 2007; 15:133–145. https://doi.org/10.1080/10673220701551102 17687708

[26] Barbui C , Esposito E, Cipriani A. Selective serotonin reuptake inhibitors and risk of suicide: a systematic review of observational studies. CMAJ. 2009; 180:291–297. https://doi.org/10.1503/cmaj.081514 19188627 PMC2630355

[27] Forte A , Buscajoni A, Fiorillo A, Pompili M, Baldessarini RJ. Suicidal risk following hospital discharge: a review. Harv Rev Psychiatry. 2019; 27:209–216. https://doi.org/10.1097/HRP.0000000000000222 31274577

[28] Tondo L , Baldessarini RJ. Prevention of suicidal behavior with lithium treatment in patients with recurrent mood disorders. Int J Bipolar Disord. 2024; 12:6–18. https://doi.org/10.1186/s40345-024-00326-x 38460088 PMC10924823

[29] Miola A , Tondo L, Pinna M, Contu M, Baldessarini RJ. Comparison of bipolar disorder type II and major depressive disorder. J Affect Disord. 2023; 323:204–212. https://doi.org/10.1016/j.jad.2022.11.039 36410453

[30] Amsterdam JD , Xu C. Multi-trial, aggregated, individual participant data mega-analysis of short-term antidepressant versus mood stabilizer monotherapy of bipolar type II major depressive episode. Bipolar Disord. 2024; 26:255–264. https://doi.org/10.1111/bdi.13378 37749069

[31] Tondo L , Baldessarini RJ, Vázquez G, Lepri B, Visioli C. Clinical responses to antidepressants among 1036 acutely depressed patients with bipolar or unipolar major affective disorders. Acta Psychiatr Scand. 2013; 127:355–364. https://doi.org/10.1111/acps.12023 23121222

[32] Kadakia A , Dembek C, Heller V, et al Efficacy and tolerability of atypical antipsychotics for acute bipolar depression: a network meta-analysis. BMC Psychiatry. 2021; 21:249–264. https://doi.org/10.1186/s12888-021-03220-3 33975574 PMC8112003

[33] Pacchiarotti I , Tiihonen J, Kotzalidis GD, et al Long-acting injectable antipsychotics (LAIs) for maintenance treatment of bipolar and schizoaffective disorders: a systematic review. Eur Neuropsychopharmacol. 2019; 29:457–470. https://doi.org/10.1016/j.euroneuro.2019.02.003 30770235

[34] Selle V , Schalkwijk S, Vázquez GH, Baldessarini R. Treatments for acute bipolar depression: meta-analyses of placebo-controlled, monotherapy trials of anticonvulsants, lithium, and second-generation antipsychotics. Pharmacopsychiatry. 2014; 47:43–52. https://doi.org/10.1055/s-0033-1363258 24549862

[35] Hirschfeld RMA , Bowden CL, Gitlin MJ, et al Practice Guideline for the Treatment of Patients with Bipolar Disorder. 2nd ed. American Psychiatric Association; 2010.

[36] Miklowitz DJ , Efthimou O, Furukawa TA, et al Adjunctive psychotherapy for bipolar disorder: a systematic review and component network meta-analysis. JAMA Psychiatry. 2021; 78:141–150. 33052390 10.1001/jamapsychiatry.2020.2993 PMC7557716

[37] Simjanoski M , Patel S, Boni R, et al Lifestyle interventions for bipolar disorders: a systematic review and meta-analysis. Neurosci Biobehav Rev. 2023; 152:105257–105269. https://doi.org/10.1016/j.neubiorev.2023.105257 37263531

